# A 48-Year-Old Woman With a History of Severe Asthma and Bilateral Minute Pulmonary Nodules

**DOI:** 10.1016/j.chpulm.2025.100198

**Published:** 2025-07-23

**Authors:** Benjamin Bottet, Nicolas Piton, Hugo Couturier, Matthieu De Meyere, Jean-Marc Baste

**Affiliations:** a Department of General and Thoracic Surgery, CHU Rouen, Université de Rouen Normandie, Rouen, France; b Department of Pathology, CHU Rouen, Université de Rouen Normandie, Rouen, France; c Department of Pneumology, CHU Rouen, Université de Rouen Normandie, Rouen, France; d Department of Radiology, CHU Rouen, Université de Rouen Normandie, Rouen, France; e Inserm U1245, Université de Rouen Normandie, Rouen, France

## Abstract

A 48-year-old woman was referred to a thoracic surgeon for lung biopsies because of the presence of widespread bilateral minute pulmonary nodules. She had been followed up and treated for nonallergic asthma since 27 years of age and sleep apnea syndrome. She was treated with high-dose inhaled corticosteroids combined with long-acting β-agonists and anticholinergic bronchodilators. For 4 years, she experienced > 3 asthma exacerbations related to air pollution and respiratory infections that required oral corticosteroid therapy. She had never smoked and kept domestic birds and rabbits for several years. She described an increase in respiratory symptoms when handling hay. Given the severity of the symptoms, her outpatient pulmonologist proposed introducing a new treatment with biologic therapy for the severe asthma. However, before initiation of therapy, a CT scan was performed for further evaluation.

## Physical Examination Findings

Height was 147 cm, and weight was stable at 72 kg (BMI, 33.3 kg/m^2^). Vital signs were normal; oxygen saturation was 95% with room air and no fever. Respiratory, cardiovascular, abdominal, and neurologic examinations revealed no abnormalities.

## Diagnostic Studies

CBC count with differential including eosinophils as well as the comprehensive metabolic panel reported findings within normal ranges. Negative results were reported for angiotensin converting enzyme and antineutrophil cytoplasm antibody precipitins. Precipitin tests showed positive results with 3 arcs of precipitation on moldy hay, but negative results for birds.

Bronchoscopy revealed no visible airway abnormalities. Bacteriologic, mycobacteriologic, and fungal samples showed negative results. Bronchoalveolar lavage found macrophagic alveolitis, without hyperlymphocytosis, and no malignant cells. The Golde score was null. Results of pulmonary function testing (with inhaled medication) were: FEV_1_, 2.42 L, 120% predicted; FVC, 3.04 L, 127% predicted; FEV_1_ to FVC ratio, 0.79; TLC, 4.52 L, 117% predicted; and diffusing capacity of the lungs for carbon monoxide, 98% predicted.

**Figure 1:**
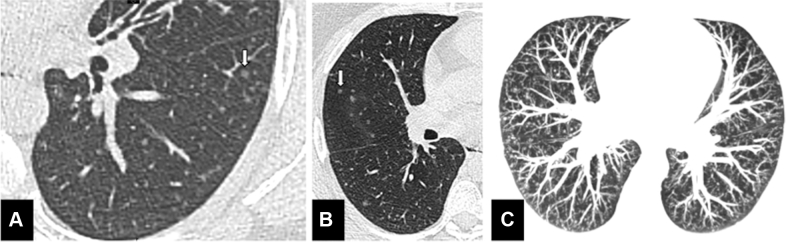
A, B, Axial chest CT scans of the lung window showing multiple diffuse subsolid micronodules without sparing of the right horizontal fissure (B) or left oblique fissure (A). Lesions marked by vertical white arrow demonstrate subtle central lucencie. C, Chest CT scans with maximum intensity projection highlighting the diffuse and randomly distributed lesions throughout both lungs.

High-resolution CT imaging of the chest revealed multiple diffuse subsolid nodules ranging from 1 to 4 mm distributed in a random pattern and demonstrating central lucencies (cheerio sign) in some cases (Fig 1). Mild air trapping and bronchial thickening were present. No evidence was found of cyst, solid nodule, nor sparing of the basal lung and the cardiophrenic angles. The mediastinum, upper kidneys, and bones in the field of view were free of any lesions.

**Figure 2:**
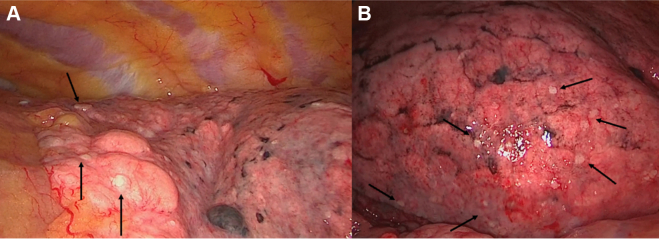
A, B, Photographs showing intraoperative views of the right lower lobe (A) and the middle lobe (B) with multiple whitish nodules (black arrows) diffusely distributed over the lung parenchyma. The parietal pleura was healthy macroscopically.

Macroscopic examination during surgery showed numerous benign-appearing diffuse millimetric nodules with a whitish appearance. The lung was free of adhesions. The mediastinal, diaphragmatic, and parietal pleura were healthy macroscopically (Fig 2). Subsequently, a surgical lung biopsy was performed on each right lobe for further evaluation and analysis by video-assisted thoracoscopic surgery.

**Figure 3:**
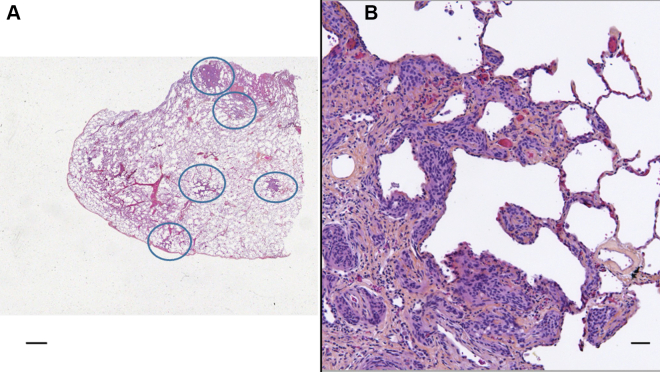
A, B, Photomicrographs showing microscopic examination of the lung, revealing multiple foci of cell proliferation with a perivenular pattern of growth and ill-defined borders (A, circles) and, at higher magnification (B), cells forming organoid nests. Cytologically, they are bland oval with a moderate amount of eosinophilic cytoplasm. Nucleoli are faint or absent. Scale bars indicate 2 mm (A) and 50 μm (B). Hematoxylin and eosin staining. Magnification x5 (A) and x20 (B).

Microscopic examination revealed multiple foci of meningothelial cell proliferation with a perivenular pattern of growth and ill-defined borders (Fig 3). They all measured < 0.3 cm, but sometimes appeared to be confluent. These meningothelial cells showed the following immunohistochemical profile: positivity for somatostatin receptor 2 (SSTR2), progesterone receptor (PR), and CD56, and negativity for protein S100 (S100), human melanoma black 45 (HMB45), estrogen receptor (ER), smooth muscle actin (SMA), ETS-related gene (ERG), CD31, melan A (MART-1), AE1/AE3, MNF116, chromogranin, synaptophysin, thyroid transcription factor-1 (TTF1), and P40. The cell proliferation index estimated by Ki67 immunostaining was low at < 5%. No granulomatous lesions were noted.

*What is the diagnosis?*

*Diagnosis*: Diffuse pulmonary meningotheliomatosis (DPM)

## Discussion

DPM is an exceptionally rare pulmonary disease of uncertain origin. It is characterized by the presence of numerous bilateral minute pulmonary meningothelial-like nodules (MPMNs) within the lung tissue. Initially described as “chemodectomas” by Korn et al in 1960 because of their close proximity to blood vessels, subsequent studies using advanced techniques, such as immunohistochemical examination, ultrastructural analysis, and fluorescence in situ hybridization, established their meningothelial nature.

MPMNs and DPM typically present as benign, asymptomatic, and incidental findings during chest CT scan or lung surgeries performed for unrelated reasons. Although these nodules can be observed across a broad age range (12-92 years), they are encountered most commonly in individuals between 50 and 70 years of age, with a notable female predominance (female to male ratio, 9:1). MPMNs can manifest in different ways, either as solitary nodules or as multiple nodules confined to a specific lobe, or may be distributed diffusely, in which case they are called DPM. Although DPM usually does not cause symptoms, in some patients, it may result in cardiorespiratory symptoms (shortness of breath, cough, and chest discomfort) and mild restrictive lung disease, as seen in certain patients. However, these manifestations often are nonspecific, leading to challenges in diagnosing the disease.

High-resolution CT imaging of the chest is essential for detecting and characterizing these pulmonary micronodules. This imaging technique not only helps to characterize nodular formations, but also plays a key role in excluding other diagnoses of alarming diseases that might present with similar micronodular manifestations.

MPMN typically appears as a rounded ground-glass opacity with clear edges and central lucencies that might be observed. Distribution of multiple MPMNs typically is random, but mild subpleural predominance can be observed. When multiple MPMNs appear and are associated with the cheerio sign, the differential diagnosis on imaging should include various neoplastic, inflammatory, or infectious diseases such as lung adenocarcinoma and metastasis, pulmonary Langerhans cell histiocytosis, granulomatosis, and fungal or mycobacterial infections.

However, the diagnosis cannot be based on the radiologic features. Histopathologic confirmation is imperative to establish the diagnosis of DPM definitively. Lung biopsies can be obtained either transparietally or surgically using video-assisted thoracoscopic surgery. Currently, minimally invasive thoracic surgery techniques and enhanced recovery after surgery programs enable us to offer patients a minimally morbid procedure, while providing pathologists with high-quality specimens.

The expression of SSTR2 by tumor cells indicates their meningothelial nature. The main differential diagnosis is meningioma of the lung, but some features are characteristic of MPMN: ill-defined borders and size usually < 3 mm and predominant interstitial or perivenular distribution.

Management of DPM primarily is conservative, involving surveillance imaging to monitor the stability of the nodules. Although DPM generally is considered benign, it is crucial to be aware of this rare entity because it can be mistaken for other disease processes, especially metastatic cancer, infections, or interstitial lung diseases.

### Clinical Course

In this patient, the main differential diagnosis was hypersensitivity pneumonitis, given the positive serum precipitins to moldy hay and the fact that sometimes she handled it to her pets. However, exposure to this allergen was nonsignificant, and bronchoalveolar lavage findings did not support this hypothesis. Other differential diagnoses were another granulomatosis resulting from sarcoidosis or infection and metastatic disease. Absence of pulmonary cyst, solid nodules, and lymph node in a somewhat healthy patient was suggestive of MPMNs. The patient was informed about the benign nature of the disease. She recovered well from the postoperative phase. The 1-year follow-up revealed no progression of the disease. Furthermore, the cerebral MRI did not identify any presence of a meningioma.

## Clinical Pearls

1. *DPM is a rare and benign disease characterized by the presence of numerous bilateral minute pulmonary meningothelial-like nodules.*
2. *DPM is observed more commonly in female patients, predominantly within the age range of 50 to 70 years.*
3. *Thoracic CT imaging is essential for characterizing nodules in DPM and eliminating differential diagnoses.*
4. *Although DPM is a benign but rare disease, other disease processes should not be overlooked.*
5. *Only the anatomopathologic examination of the nodules, from lung samples, can make a definitive diagnosis of DPM.*
6. *Management of DPM is primarily conservative, involving surveillance imaging to monitor the stability of the nodules.*

## Funding/Support

The authors have reported to *CHEST Pulmonary* that no funding was received for this study.

## Financial/Nonfinancial Disclosures

None declared.
